# The Asian Fabry Cardiomyopathy High-Risk Screening Study 2 (ASIAN-FAME-2): Prevalence of Fabry Disease in Patients with Left Ventricular Hypertrophy

**DOI:** 10.3390/jcm13133896

**Published:** 2024-07-02

**Authors:** Sophia Po-Yee Leung, Scott Dougherty, Xiao-Yu Zhang, Kevin K. H. Kam, Wai-Kin Chi, Joseph Y. S. Chan, Erik Fung, Jeffrey K. T. Wong, Paul C. L. Choi, David K. H. Chan, Bun Sheng, Alex Pui-Wai Lee

**Affiliations:** 1 Laboratory of Cardiac Imaging and 3D Printing, Li Ka Shing Institute of Health Science, The Chinese University of Hong Kong, Hong Kong, China; 1155182334@link.cuhk.edu.hk (S.P.-Y.L.); hydzxy@126.com (X.-Y.Z.); 2 Department of Cardiology, Tseung Kwan O Hospital, Hong Kong, China; sdd461@ha.org.hk; 3 Division of Cardiology, Department of Medicine and Therapeutics, The Chinese University of Hong Kong, Hong Kong, China; kkh381@ha.org.hk (K.K.H.K.); cwk743@ha.org.hk (W.-K.C.); cys644@ha.org.hk (J.Y.S.C.); e.fung@cuhk.edu.hk (E.F.); 4 Department of Imaging and Interventional Radiology, The Chinese University of Hong Kong, Hong Kong, China; wongkatakjeffrey@hotmail.com; 5 Department of Anatomical and Cellular Pathology, The Chinese University of Hong Kong, Hong Kong, China; paulchoi@cuhk.edu.hk; 6 Elderly Health Service, 11/F, ChinaChem Exchange Square, 1 Hoi Wan St, Quarry Bay, Hong Kong, China; davidchan68@hotmail.com; 7 Princess Margaret Hospital, 2-10 Princess Margaret Hospital Road, Kwai Chung, Hong Kong, China; shengb@ha.org.hk

**Keywords:** Fabry disease, lysosomal storage disorder, rare disease, left ventricular hypertrophy, genetics, late-onset cardiac variant

## Abstract

**Background:** Fabry disease (FD) is a rare X-linked lysosomal storage disorder that commonly manifests cardiovascular complications. We aimed to assess the prevalence of FD in a Chinese population with left ventricular hypertrophy (LVH) whilst implementing a gender-specific screening approach. **Methods:** Patients with LVH, defined as a maximum thickness of the left ventricular septal/posterior wall ≥ 13 mm, were considered eligible. All patients with hypertrophic cardiomyopathy (HCM) were excluded. Plasma α-galactosidase (α-GLA) enzyme activity was assessed using a dried blood spot test. Males with low enzyme activity underwent genetic testing to confirm a diagnosis of FD whereas females were screened for both α-GLA and globotriaosylsphingosine concentration and underwent genetic analysis of the GLA gene only if testing positive for ≥1 parameter. **Results:** 426 unrelated patients (age = 64.6 ± 13.0 years; female: male = 113:313) were evaluated. FD was diagnosed in 3 unrelated patients (age = 69.0 ± 3.5 years, female: male = 1:2) and 1 related female subject (age = 43 years). Genetic analyses confirmed the late-onset cardiac variant GLA c.640-801G>A (*n* = 3) and the missense variant c.869T>C associated with classic FD (*n* = 1). Cardiac complications were the only significant findings associated with the late-onset c.640-801G>A mutation, manifesting as mild or severe concentric LVH. In contrast, the classic c.869T>C mutation FD exhibited multisystemic manifestations in addition to severe concentric LVH. **Conclusions:** The prevalence of FD is lower in Chinese patients with LVH when HCM is excluded. The pathological variant c.640-801G>A remains the most common cause of late-onset FD, while the detection of FD in females can be improved by utilizing a gender-specific screening method.

## 1. Introduction

Fabry disease (FD) is a rare X-linked lysosomal storage disorder caused by mutations in the GLA gene resulting in deficient or absent α-galactosidase A (α-GLA) enzyme activity. FD is characterized by progressive accumulation of globotriaosylceramide (Gb3) and other glycosphingolipids throughout the body, leading to multisystem involvement and major organ dysfunction including cardiac, renal, and cerebrovascular disease [1].

The estimated prevalence of FD ranges from 1:40,000 to 1:117,000 [2], which is likely an underestimation due to the non-specific manifestations of FD and low clinical suspicion of the disease. Indeed, evidence suggests that FD is increasingly recognized but remains under-diagnosed [3,4,5]. Newborn screening initiatives suggest the prevalence of FD could be as high as 1 in 1250 in Taiwanese males, 1 in 4600 in Italy [6], and 1 in 7057 in Japan [7]. The prevalence of FD in adult populations with hypertrophic cardiomyopathy (HCM) or left ventricular hypertrophy (LVH) is most commonly 0–4% [8]. Our recent study (ASIAN-FAME) revealed that the prevalence of FD among Hong Kong Chinese patients with HCM or LVH was 1.6% in the overall population (8/499) and 2.4% in males (8/336) [9].

Over recent decades, the clinical spectrum of FD has also expanded from the “classic” phenotype—which presents in childhood with cutaneous, gastrointestinal, and neurological symptoms—to include a late-onset phenotype which typically manifests from the third to fourth decades onwards and is characterized by LVH and cardiac symptoms. In Chinese patients, this late-onset cardiac variant is most often associated with the GLA c.640-801G>A mutation (traditionally known in the literature as IVS4+919G>A) [10] and was found in all of our confirmed FD patients in the ASIAN-FAME study [9]. Ongoing research in other populations continues to identify other variants that are also linked to this late-onset variant, including p.Asn215Ser (p.N215S), T410A/GLA, and GLA c.337T>C (p.Phe113Leu) [11,12,13,14].

Previous FD screening studies enrolled patients based on a diagnosis of either HCM or LVH [15,16,17,18,19,20,21,22,23,24,25,26,27,28,29,30,31,32,33,34,35,36,37,38,39,40,41]. This approach optimizes case detection given that FD can masquerade as HCM—therefore some “HCM” cases would be expected to be subsequently reclassified as FD. Conversely, many of these screening studies excluded patients with LVH and concomitant hypertension or aortic stenosis, which would likely miss some FD cases given that the cause of LVH in these patients may be multifactorial. Another weakness of these screening studies is that some only enrolled male subjects, whilst those that did recruit both sexes did not use screening methods that enhanced female case detection.

Due to a lack of systematic screening, the true prevalence of FD among Hong Kong Chinese patients with LVH remains unknown. In particular, genetic testing is rarely conducted in patients with unexplained LVH, partly because of the lack of dedicated local cardiomyopathy centers. Therefore, in the present ASIAN-FAME-2 study, we aim to extend our previous investigations to evaluate the prevalence of FD among unselected Chinese patients of both sexes with LVH. We hypothesized that we would reveal undiagnosed FD, and that gender-specific screening can improve FD detection in females.

## 2. Methods

### 2.1. Study Population

This observational cross-sectional study was conducted at a tertiary university hospital in Hong Kong. From March 2020 to August 2022, a total of 426 adult (aged ≥18 years) consecutive patients with LVH were recruited for FD screening if they had maximal left ventricular (septal and/or posterior) wall thickness ≥13 mm, as determined by transthoracic echocardiography. Patients with abnormal loading conditions, namely hypertension and aortic stenosis, were included in the study. Exclusion criteria included those with a diagnosis of HCM (i.e., increased LV wall thickness of ≥13 mm not solely explained by abnormal loading conditions or those with known sarcomere mutations), other infiltrative cardiomyopathies (e.g., cardiac amyloidosis), LV non-compaction, athlete’s heart, morbid obesity, and other non-FD infiltrative cardiomyopathies associated with LVH. The study adhered to the principles outlined in the Declaration of Helsinki and received approval from the institution’s human research committee. Written informed consent was obtained from all participants.

### 2.2. FD Screening Process

**Figure 1:**
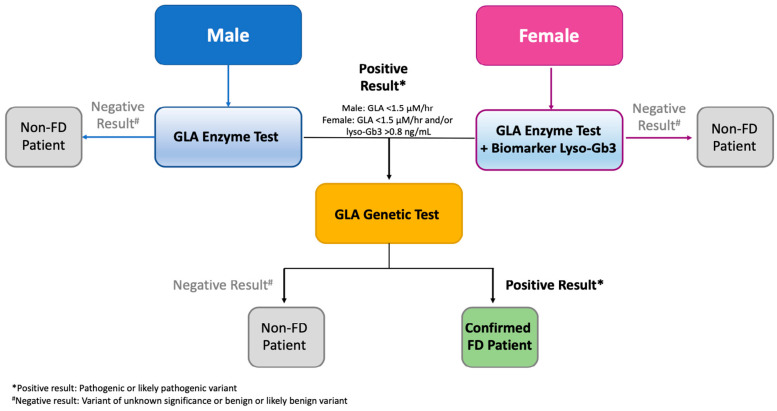
Flow chart of Fabry disease patient screening process. FD = fabry disease; GLA = alpha-galactosidase A.

To measure α-GLA enzyme activity in the blood samples of all patients, a dried-blood spot (DBS) assay was conducted using the MS/MS method [42], as previously described by the biochemical lab at the National Taiwan University Hospital [43]. The control enzyme, alpha-L-iduronidase (IDUA), was utilized in duplication. For female subjects, in addition to α-GLA enzyme activity, lyso-Gb3 concentrations were analyzed in DBS samples following extraction using an HPLC-mass spectrometry (MS)/MS method [44]. Different screening protocols were implemented for males and females (Figure 1). Males underwent primary screening by assessing T-lymphocyte α-GLA enzyme activity. Subsequently, individuals with low enzyme activity underwent genetic testing as a confirmatory measure for FD. Females underwent screening for both T-lymphocyte α-GLA enzyme activity and lyso-Gb3 concentration. If they tested positive for at least one of these parameters, genetic testing was conducted. In males, FD was suspected when α-GLA activity was below 1.5 µM/h, while in females, FD was suspected when α-GLA activity was below 1.5 µM/h or lyso-Gb3 concentration exceeded 0.8 ng/mL [45]. For patients with low plasma α-GLA activity (defined as an IDUA/GLA ratio ≥ 10) [43] and/or lyso-Gb3>0.8 ng/mL, 3 mL of blood was collected and kept in an ethylenediaminetetraacetic acid tube for GLA gene sequencing using the Sanger method. Starting from April 2022, α-GLA activity, lyso-Gb3 concentration, and GLA gene sequencing were performed through a single DBS test.

### 2.3. Demographics and Data Collection

Demographic data, patients’ history, and laboratory results were reviewed from their medical charts. Chinese ethnicity subgroups (Teochew, Canton, Fujian, Shanghai, Others, and Unknown) were self-reported by the subjects. First-degree relatives of each genetically confirmed FD patient were invited to undergo familial screening.

### 2.4. Cardiac Evaluation

Standard two-dimensional (2D) and Doppler transthoracic echocardiography was performed on all subjects. Standard 2D measurements were obtained as recommended by the American Society of Echocardiography [46]. LV ejection fraction (LVEF) was calculated using Simpson’s biplane method. LV mass was calculated by the linear method and indexed to body surface area. The relative wall thickness (RWT) was calculated with the formula (2 × end-diastolic posterior wall thickness)/LV end-diastolic dimension. All measurements were made in 3 cardiac cycles in sinus rhythm and 5 cardiac cycles in patients with atrial fibrillation. Endomyocardial biopsy was offered for all genetically confirmed FD patients for whom treatment with enzyme replacement therapy (ERT) was planned, as required per protocol by the Hong Kong government before treatment subsidization can be offered.

### 2.5. Statistical Analyses

Data are shown as mean ± standard deviation, range, or number (percentage). Comparisons between FD and non-FD patients were made using Fisher’s exact test, chi-square test, or Student’s *t*-test, where appropriate. A *p*-value of <0.05 was considered statistically significant. All statistical analyses were performed using SPSS 29.0 (IBM Corp., Armonk, NY, USA).

## 3. Results

### 3.1. Study Population and Genetic Screening

Table 1 provides a comprehensive overview of the patient characteristics, clinical features, and echocardiographic findings observed in the study. A total of 426 individuals participated, consisting of 313 males and 113 females, with an average age of 65 ± 13 years (ranging from 18 to 90 years). The overall interventricular septal thickness at end-diastole (IVSd) measured 15.2 ± 2.2 mm, while the left ventricular posterior wall thickness at end-diastole (LVPWd) measured 13.1 ± 2.5 mm. Additionally, the overall left ventricular mass index (LVMI) was calculated to be 131.9 ± 38.9 g/m^2^.

Following genetic analysis, three unrelated patients (Patients 1, 2, and 3) were identified. Patient 1 and Patient 2 were confirmed to have the late-onset cardiac variant c.640-801G>A, while Patient 3 exhibited the classic FD missense variant c.869T>C (p.Met290Thr) (Table 2). Consequently, the overall prevalence of FD in the population was 0.7% (3 out of 426 individuals), with a prevalence of 0.6% in men and 0.9% in women. Among the FD patients, the mean age was 69.0 ± 3.5 years, with an average IVSd of 20.7 ± 5.7 mm (*p* = 0.019), LVPWd of 16 ± 5.0 (*p* = 0.241), and an LVMI of 221 ± 61.6 g/m^2^ (*p* = 0.012).

### 3.2. Fabry Disease Patients

All FD patients reported their ethnicity as Canton. Common clinical manifestations among these patients included hypertension (*n* = 3), mitral regurgitation (*n* = 1), impaired estimated glomerular filtration rate (eGFR) below 60 mL/min/1.73 m^2^ (*n* = 1), heart failure (*n* = 1), atrial fibrillation (*n* = 1), and proteinuria (*n* = 1). Neither Patient 1 nor Patient 2, both of whom had the late-onset cardiac variant (c.640-801G>A), exhibited classic neurological, ophthalmic, dermatological, or other typical FD symptoms. In these patients, the heart was the only clinically affected organ, characterized by LVH (Patient 2 had heart failure but this was related to rheumatic heart disease and not FD). In contrast, Patient 3 had classic FD and presented with involvement of multiple systems, including hypohidrosis, gastrointestinal symptoms, acroparesthesia, hearing loss, and severe concentric LVH.

ECG features of FD include the Sokolow-Lyon index, the ratio between the P-wave and PR-segment duration, QRS duration, and QT duration, and were observed in some of our FD patients. The ECG of Patient 1 showed a ventricular paced rhythm, which limited interpretation and there were no FD features on the ECG of Patient 4. However, for Patient 2 we observed right bundle branch block and Patient 3 met LVH criteria using the Sokolow-Lyon index. Other ECG features of FD, including shortened P-waves, lower T-wave amplitude, and a lower (T_onset_ − T_peak_)/(T_peak_ − T_end_) ratio were also not observed in our cohort [47,48].

### 3.3. Patient 1

A 72-year-old man with a known history of poorly-controlled hypertension (diagnosed in his 30 s but did not receive treatment until his 50 s) and chronic kidney disease secondary to IgA nephropathy and hypertensive nephrosclerosis (post cadaveric renal transplant in 2001). Presented in 2009 with syncope and subsequently labelled “obstructive HCM” based on echocardiogram. He then had a DDDR pacemaker and alcohol septal ablation in 2020 with improvement in dizziness. He underwent FD screening in 2020 in view of severe LVH which showed α-GLA activity of 1.37 μM/h (normal > 1.5 μM/h). Genetic analysis found the c.640-801G>A late-onset pathologic variant. CMR showed diffuse LV wall thickening, increased myocardial mass (191.07 g/m^2^) and mild diffuse RV wall thickening. T1 and T2 mapping were normal. Transmural late gadolinium enhancement (LGE) at the basal septal segment and diffuse mottled mid-wall LGE at the basal and mid-ventricular lateral walls were noted, suggestive of non-ischemic myocardial fibrosis. He started enzyme replacement therapy (ERT) in July 2022 with improved exercise tolerance (can now manage 2 flights of stairs).

### 3.4. Patient 2

A 71-year-old man with a known history of hypertension, atrial fibrillation (AF), rheumatic heart disease (RHD), and LVH. He was first diagnosed with RHD in 1995 and LVH in 1999. Echo in 2018 showed severe LVH, severe MR, and ruptured chordae requiring mitral valve repair and tricuspid annuloplasty in 2021. In 2021 he also underwent screening for FD given the severe LVH. His α-GLA activity was 0.82 μM/h (normal > 1.5 μM/h). CMR showed concentric LV wall thickening, mildly impaired LVEF (45%) and mild LV dilatation. No regional LV wall motion abnormality was observed. Non-ischemic mid-wall myocardial LGE at the basal and mid ventricular LV lateral walls revealed myocardial fibrosis related to FD. Due to recurrent MR, he then required valve-in-annuloplasty transcatheter mitral valve replacement in July 2023. The patient had planned to initiate ERT after valvular surgery.

### 3.5. Patient 3

A 65-year-old woman with a past history of hypertension and LVH first diagnosed by ECG in 2011. Since her 60s she reported profound hearing loss and more recently experienced hypohidrosis and gastrointestinal symptoms. She also reported bilateral hand numbness which was also Tinel’s positive. She underwent a formal body check in 2021 including an echocardiogram which showed severe LVH. Her α-GLA activity was 2.88 μM/h (normal >1.5 μM/h) and Lyso-Gb3 10.08 ng/mL (normal < 0.80 ng/mL). Genetic analysis revealed she carried the classic variant c.869T>C (p.Met290Thr). CMR revealed mild LV wall thickening, shortening of T1 relaxation time on T1 mapping and no LGE was present. The patient has not yet started ERT yet because she is indecisive about undergoing endomyocardial biopsy, which is a requirement for government funded ERT.

### 3.6. Endomyocardial Biopsy

**Figure 2:**
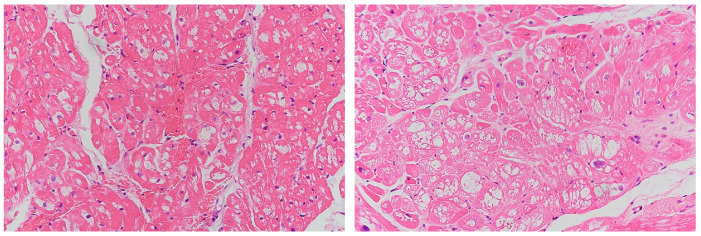
Histologic examination of FD patients with c.640-801G>A mutation. Hematoxylin-eosin staining showed vacuolated myocardial cells (**Left**: Patient 1; **Right**: Patient 2).

Endomyocardial biopsy was performed for Patient 1 and Patient 2 with the c.640-801G>A mutation. Under microscopic examination, cardiomyocytes showed cytoplasmic vacuolation and myocardial tissue with focal fibrosis (Figure 2). Abundant lamellated myelin bodies were found on electron microscopy, similar to the findings of previous reports regarding late-onset FD. For Patient 3, endomyocardial biopsy was not performed due to patient reluctance.

### 3.7. Family Screening

**Figure 3:**
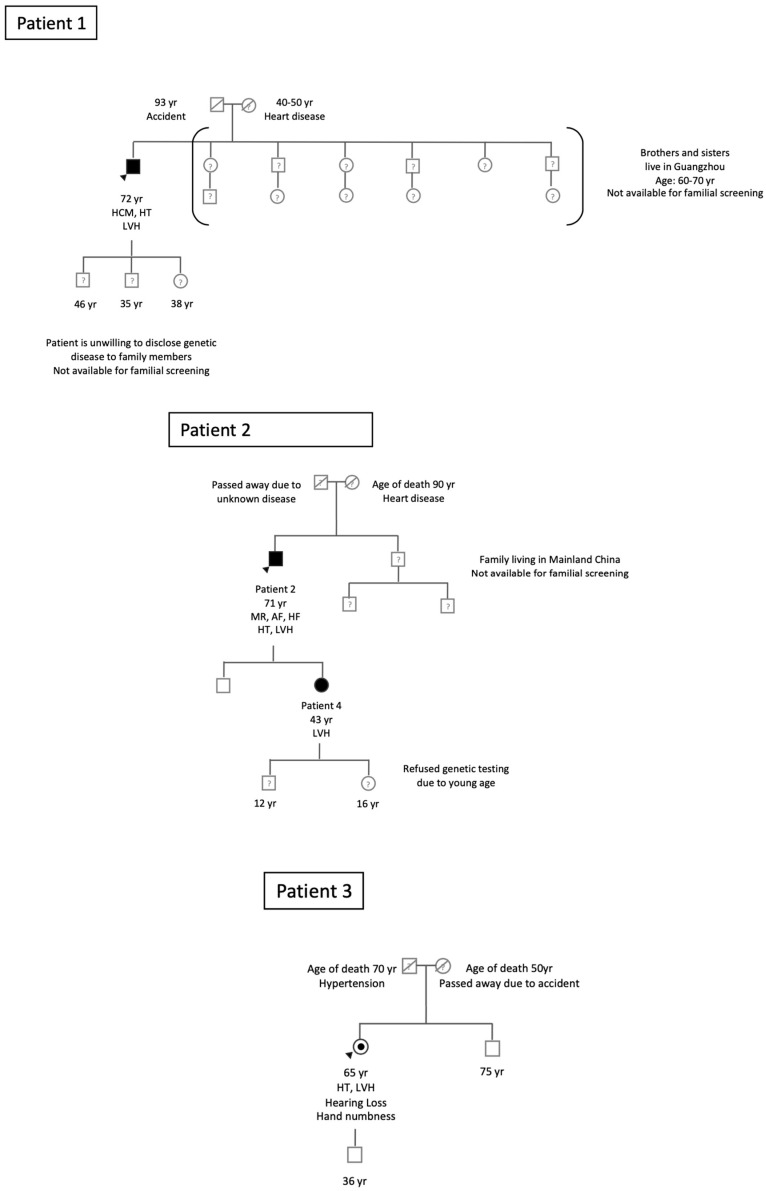

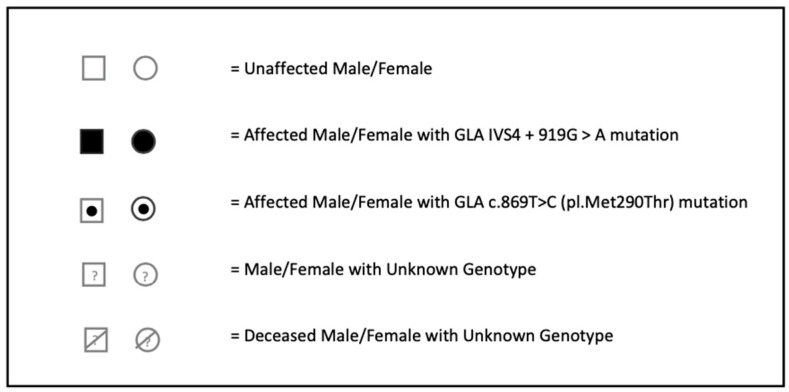
Pedigree of Index Fabry Patients 1–3. Circles are women and squares are men. Empty symbols are unaffected relatives without mutation. Filled symbols are affected patients with the GLA IVS4+919G>A mutation. Dotted symbols are affected patients with the GLA c.869T>C (pl.Met290Thr) mutation. Question marks indicate family members with an unknown genotype as a genetic test was not performed. Dashed symbols are deceased family members. Numbers are ages. Index patients are indicated by arrow heads. AF = atrial fibrillation; HCM = hypertrophic cardiomyopathy; HF = heart failure; HT = hypertension; LVH = left ventricular hypertrophy; MR = mitral regurgitation; Yr = year.

Each FD patient with a confirmed genetic diagnosis was encouraged to involve their relatives for family screening. In one case, a related female individual (Patient 4, related to Patient 2) was identified and confirmed to have FD, as depicted in Figure 3 (pedigree tree).

For Patient 1, his father had passed away in an accident, and his mother had succumbed to heart disease in her 40 s or 50 s. Patient 1 chose not to disclose his genetic condition to his family members, resulting in none of them (including 3 brothers, 3 sisters, 2 sons, and 1 daughter) participating in familial screening. However, it is likely that his 38-year-old daughter has inherited FD given that its inheritance is X-linked.

As for Patient 2, his father’s cause of death remains unknown, while his mother passed away from heart disease at the age of 90. Both his son and daughter consented to screening, but his younger brother’s two sons, residing in Mainland China, were unable to participate. The screening results showed that his son tested negative for FD; however, his daughter (Patient 4, aged 43) was confirmed to carry the late-onset cardiac variant c.640-801G>A, as indicated in Table 2. Patient 4 has a son and a daughter, but she declined screening for her children due to their young age (12 and 16 years old).

Patient 3’s father had passed away at the age of 70 due to hypertension, and her mother died in an accident. Both her son and elder brother tested negative for FD.

## 4. Discussion

In the ASIAN-FAME-2 study, the prevalence of FD in our non-selective LVH population was 0.7%, which is consistent with previous FD echocardiographic screening studies that found a prevalence of 0–4% [8]. We also found that most (2 out of 3) of our positive FD patients had the late-onset pathologic variant c.640-801G>A, which also is consistent with previous studies.

To overcome important limitations of previous screening studies [11,12,13,14,15,16,17,18,19,20,21,22,23,24,25,26,27,28,29,30,31,32,33,34,35,36,37], our study broadened the inclusion criteria and diagnostic methodology in order to improve diagnostic sensitivity, particularly in females. First, we included both sexes, which contrasts to many studies in which only males were included. Second, we enrolled patients with LVH and concomitant hypertension and aortic stenosis. Finally, we utilized a gender-specific screening method to improve the diagnostic detection of FD in females.

Whilst α-GLA enzyme activity assessment is an effective screening tool for FD in males, it may not reliably detect FD in heterozygous females because they have variable levels of α-GLA activity due to random X-chromosome inactivation. Previous studies have shown that using enzyme activity alone for determination of classic FD in females results in a 40% false-negative rate [49]. In our experience, however, patients (regardless of sex) with the late-onset cardiac variant c.640-801G>A, can be effectively detected by α-GLA enzyme testing alone with a false-negative rate of 0% [9,34]. Additional screening for lyso-Gb3 levels therefore is intended for improved detection of female patients with classic FD. Indeed, our positive female case with classic FD had normal α-GLA enzyme activity but an elevated lyso-Gb3 assay, suggesting that our gender-specific methodology may be more effective at detecting FD in women with classic FD, but larger studies are required to evaluate this further.

We also excluded HCM patients for the purpose of evaluating FD prevalence in LVH patients commonly seen in daily clinical practice, including those with hypertension and aortic stenosis. To the best of our knowledge, ours is the first study to adopt this approach, in combination with gender-specific screening. Excluding patients with HCM likely helps explain the lower prevalence of FD in our study compared to ASIAN-FAME [9] (0.7% and 1.6%, respectively), which enrolled HCM patients (making up 47% of the overall population).

Although HCM is caused by mutations in genes that encode sarcomeric proteins or sarcomere-related structures, the clinical definition of HCM is predicated on imaging evidence of increased LV wall thickness that is not explained solely by abnormal loading conditions. We know however from pathologic analyses that a significant minority of clinically diagnosed HCM cases are caused by alternative pathologies relating to inborn errors of metabolism and metabolic storage disorders. These conditions, known as HCM phenocopies, include FD, which accounts for approximately 0.3-5% of patients labelled as HCM [16,20,33]. Therefore, excluding all HCM patients from our study has also likely excluded some FD patients.

Another potential reason for the lower prevalence of FD in our study in relation to ASIAN-FAME [9] is that in the latter study, a disproportionate number of positive cases (62.5%) were of Teochew ethnicity, despite only representing 17% of the overall study cohort. The remaining cases were largely of Canton origin (25%). In comparison, our study only enrolled about a quarter of those who identified as Teochew (4.2%) compared to ASIAN-FAME [9] and all of our positive cases were of Canton origin. This suggests that the relative proportion of each ethnic subgroup may alter FD prevalence, even whilst testing in an Asian hot-spot population.

Our results add to the emerging epidemiologic profile of FD, underscoring the importance of including FD in the differential diagnosis of LVH, including in those patients with concomitant hypertension and aortic stenosis. However, given the impracticalities and cost implications of screening all patients with LVH for FD, particularly in resource-limited settings, assimilation of key echocardiographic data and clinical red flags for FD, as defined in the 2023 European Society of Cardiology guidelines on cardiomyopathies [50], may provide a practical basis for identifying which patients should be screened for FD in clinical practice. Additionally, it is worth considering targeting FD screening to patients with LVH and certain high-risk features (as observed in the ASIAN-FAME study [9]), such as patients with concomitant heart failure or history of renal transplant.

There are also no controlled studies demonstrating a morbidity or mortality benefit of treating screening-detected FD compared to clinically-detected FD. Moreover, whilst the efficacy of ERT in treating classic FD has an established evidence base, there is very limited data demonstrating a similar benefit in the late-onset cardiac variant. The cost of ERT (approximately 1 million HKD per year locally) is currently prohibitive for the majority of patients in our local population, most of whom rely on government subsidies to facilitate treatment. Addressing these knowledge gaps is therefore important when considering the role of FD screening and treatment beyond the research arena and into the wider clinical population if such significant expenditure is to be justified.

## 5. Study Limitations

Our study has several limitations. First, our screening methodology was predicated on detecting reduced α-GLA activity in males, or reduced α-GLA activity and/or increased lyso-Gb3 concentrations in females. We acknowledge that other cardiac variants of FD manifest proportionally higher α-GLA activity and that these cases may have remained undetected in our study [12,51,52]. However, although genetic and/or lyso-Gb3 screening of all participants may have provided greater sensitivity, such an approach would not have been practical or cost effective, which are also important considerations for a screening study. Second, data such as CMR and endomyocardial biopsy were not available for all FD patients during the period of manuscript preparation. Third, some FD patients’ family members were unavailable to participate in familial screening, which prevented us from completely reporting follow-up with the patients and families and arranging relevant further screening and genetic counselling.

Another important limitation is that the cut-points used in the guidelines to define LVH are based on western cohorts. It is well-established that compared to Caucasians, and even after accounting for their smaller body size, Asians have a smaller LV mass. In HCM patients, Asians have an absolute wall thickness of about 2 mm less than that reported in Caucasians [53]. Therefore, using the ≥13 mm threshold for LVH in our Asian cohort may optimize for specificity but may also compromise sensitivity, thus missing a significant number of subjects for potential screening.

## 6. Conclusions

The prevalence of FD in Hong Kong Chinese non-HCM patients with LVH was 0.7%. The late-onset cardiac variant c.640-801G>A (IVS4+919G>A) was most prevalent in this population, consistent with our previous findings. The detection of females with the classic mutation may be better achieved through the utilization of a gender-specific methodology.

## Figures and Tables

**Table 1:** Characteristics of the Overall Population and Comparisons Between Confirmed FD and Non-FD Patients.

Patient Characteristics	Overall (*n* = 426)	FD (*n* = 3)	Non-FD (*n* = 423)	*p* Value
Age (years)	64.6 ± 13.0	69.0 ± 3.5	64.6 ± 13.1	0.580
Men, *n* (%)	313 (73.0%)	2 (66.7%)	311 (73.5%)	1.000
Ethnicity subgroup, *n* (%)				1.000
Canton	332 (78.0%)	3 (100%)	329 (77.8%)	
Fujian	21 (4.9%)	0 (0%)	21 (5.0%)	
Teochew	18 (4.2%)	0 (0%)	18 (4.3%)	
Shanghai	6 (1.4%)	0 (0%)	6 (1.4%)	
Others	11 (2.6%)	0 (0%)	11 (2.6%)	
Unknown	38 (8.9%)	0 (0%)	38 (9.0%)	
Hypertension, *n* (%)	257 (60.3%)	3 (100%)	254 (59.6%)	0.280
Heart failure, *n* (%)	53 (12.4%)	1 (33.3%)	52 (12.3%)	0.329
Impaired eGFR (<60 mL/min/1.73 m^2^), *n* (%)	116 (27.2%)	1 (33.3%)	115 (27.2%)	1.000
Atrial fibrillation, *n* (%)	61 (14.3%)	1 (33.3%)	60 (14.2%)	0.372
Diabetes mellitus, *n* (%)	102 (23.9%)	0 (0%)	102(24.1%)	1.000
Arrhythmias, *n* (%)	34 (8.0%)	0 (0%)	34 (8.0%)	1.000
Coarctation of the aorta, *n* (%)	27 (6.3%)	0 (0%)	27 (6.4%)	1.000
Aortic stenosis, *n* (%)	19 (4.5%)	0 (0%)	19 (4.5%)	1.000
End-stage renal disease, *n* (%)	20 (4.7%)	0 (0%)	20 (4.7%)	1.000
Proteinuria, *n* (%)	16 (3.7%)	1 (33.3%)	15 (3.6%)	0.109
Short PR (<120 ms), *n* (%)	4 (0.9%)	0 (0%)	4 (1.0%)	1.000
Renal transplant, *n* (%)	5 (1.2%)	1 (33.3%)	4 (1.0%)	0.035
Uncontrolled hypertension, *n* (%)	3 (0.7%)	0 (0%)	3 (0.7%)	1.000
IVSd (mm)	15.2 ± 2.2	20.7 ± 5.7	15.1 ± 2.1	0.019
LVPWd (mm)	13.1 ± 2.5	16.0 ± 5.0	13.1 ± 2.5	0.241
LVIDd (mm)	42.0 ± 6.5	41.3 ± 4.9	42.0 ± 6.5	0.520
LVIDs (mm)	28.3 ± 5.9	28.3 ± 4.2	28.3 ± 5.9	0.951
LVEF (%)	57.9 ± 7.7	52.7 ± 12.5	58.0 ± 7.6	0.424
LVM (g)	231.7 ± 74.7	351.8 ± 158.9	230.9 ± 73.4	0.134
LVMI (g/m^2^)	131.9 ± 38.9	221.0 ± 61.6	131.3 ± 38.1	0.012
RWT	0.64 ± 0.2	0.78 ± 0.3	0.64 ± 0.16	0.255

eGFR = estimated glomerular filtration rate based on CKD-EPI equation; FD = fabry disease; IVSd = interventricular septal thickness at end-diastole; LVPWd = left ventricular posterior wall thickness at end-diastole; LVIDd = left ventricular internal diameter at end-diastole; LVIDs = left ventricular internal diameter at end-systole; LVEF = left ventricular ejection fraction; LVM = left ventricular mass; LVMI, left ventricular mass index; RWT = relative wall thickness.

**Table 2:** Characteristics of Patients with Fabry Disease.

	Patient 1	Patient 2	Patient 3	Patient 4
Age of Diagnosis of FD, years	72	71	65	43
Gender	Male	Male	Female	Female
Ethnicity Subgroup	Guangzhou	Guangdong Zhongshan	Nanhai	Guangdong Zhongshan
Medical History	- Hypertension since 30s - IgA nephropathy and hypertensive nephrosclerosis (biopsy proven and unrelated to FD) - Cadaveric kidney transplant in 2001 - Labelled “HCM” with dynamic LVOTO - DDDR pacemaker implanted in 2020 for syncope followed by alcohol septal ablation	- Rheumatic heart disease complicated by ruptured chordae, MV prolapse, and severe MR with heart failure - Open MV repair and tricuspid annuloplasty 2021 - ViR TMVR 2023 for recurrent MR - Atrial fibrillation - Hypertension	- Hypertension - LVH with strain pattern on ECG since 2011 - Deafness since 60 years old - Kyphoscoliosis	- Right parotidectomy in 2000 - Menorrhagia
Initial Presentation	- Syncope with subsequent diagnosis of “HCM”	- Heart failure secondary to underlying rheumatic heart disease and severe mitral regurgitation, echo revealed incidental severe LVH	- Incidental finding of LVH on ECG in 2011 - Mild reduction in exercise tolerance over the subsequent years - Bilateral hand numbness for >1 year prior to FD diagnosis, Tinel’s+ve - Echo for body check in 2021 showed severe LVH 2021	- Detected from cascade screening (daughter of proband patient #3) - No classical Fabry neurological symptoms - Vague SOBOE on exertion for 4–5 years (sedentary lifestyle) - No orthopnoea or paroxysmal nocturnal dyspnoea - No ankle edema
CMR	- Impaired LVEF (45.3%) - Increased LV wall thickening (IVS 12 mm, IW 20 mm) and increased myocardial mass (191 g/m^2^) - Subaortic outflow obstruction - Mild diffuse RV wall thickening - T1 & T2 mapping normal - Transmural LGE at basal septal segment, diffuse mottled mid-wall LGE basal and mid-ventricular lateral wall	- Impaired LVEF (45.3%) with mild LV dilation - Mild concentric LVH (IVS 16 mm, IW 13 mm) - No significant mitral and tricuspid dysfunction - Non-ischemic mid-wall myocardial LGE at the basal and mid ventricular LV lateral walls - RV normal size and both atria normal size	- Normal LVEF (77%) - Mild LVH (IVS 13.1 mm, IW 8.3 mm) - No significant valvulopathy - RV and both atria normal size - Mild shortening of T1 relaxation time on T1 mapping - No LGE to suggest scarring or fibrosis - No regional LV wall motion abnormality	NA
CT/MRI Brain	Bilateral lacunar infarction at capsular region	Non-specific subcortical and periventricular white matter T2W/FLAIR hyperintense foci in bilateral frontal and parietal lobes	Silent lacunar infarct over right side	NA
Echocardiogram	- Normal LVEF 65% - Severe concentric LVH (3.1 cm ateroseptum) - Peak LVOT gradient 27 mmHg (rest) and 39 mmHg (Valsalva) - Intermittent SAM with mild eccentric MR - Grade I diastolic dysfunction - RV normal size and function	Pre-op - LVEF 40% - Severe concentric LVH - Severe MR with ruptured chordae	- LVEF 53%, GLS -10% - Impaired LV relaxation - Severe concentric LVH - Mild MR and TR	- LVEF 61%, GLS -17.6% - Mild concentric LVH - No regional wall motion abnormalities - Left ventricle normal in size
Endomyocardial Biopsy and electron microscopy	- Myocardial tissue with cytoplasmic vacuolation - No increased fibrosis - Abundant lamellated myelin bodies	- Myocardial tissue with focal fibrosis - Cardiomyocytes show cytoplasmic vacuolation - Zebra bodies found in the cardiomyocytes	NA	NA
ECG	- Ventricular paced rhythm	- AF with RBBB	- SR, LVH with strain pattern	- SR, no LVH by voltage
IVSd, mm	27	19	16	11
LVPWd, mm	21	16	11	10
LVEF (%)	65	40	53	61
LVIDd, mm	39	47	38	46
LVIDs, mm	25	33	27	30
LVM, g	499	373	183	164
LVMI, g/m^2^	284	214	160	86.8
RWT	1.08	0.68	0.58	0.44
α-GLA activity, µmol/hr	1.37	0.82	2.88	0.99
LysoGb3, ng/mL	NA	NA	10.08	0.60
Creatinine, µmol/L	108	96	69	NA
eGFR, mL/min/1.73 m^2^	59	70	84	NA
24 h urinary protein (mg)	NA	160	80	NA
Protein/Cr, urine ratio	0.22	0.12	NA	NA
Albumin/Cr ratio	NA	5.1	17.8	NA
eGFR, mL/min/1.73 m^2^	59	70	84	NA
GLA Gene Mutation	c.640-801G>A	c.640-801G>A	c.869T>C (pl.Met290Thr)	c.640-801G>A
Type of FD	Late-onset	Late-onset	Classic	Late-onset
ERT Initiation Status	Started in July 2022	Not started	Not started	Not started

AF = atrial fibrillation; CMR = cardiovascular magnetic resonance; CT = computed tomography; DDDR = dual-chamber rate-modulated; ECG = electrocardiogram; eGFR = estimated glomerular filtration rate based on CKD-EPI equation; ERT = enzyme replacement therapy; FD = Fabry disease; HCM = hypertrophic cardiomyopathy; IVSd = interventricular septal thickness; LGE = late gadolinium enhancement; LV = left ventricular; LVH = left ventricular hypertrophy; LVOTO = left ventricular outflow tract obstruction; LVPWd = left ventricular posterior wall thickness; LVIDd = left ventricular internal diameter end distole; LVIDs = left ventricular internal diameter end systole; LVEF = left ventricular ejection fraction; LVM = left ventricular mass; LVMI = left ventricular mass index; MR = mitral regurgitation; MRI = magnetic resonance imaging; NA = not available; NYHA = New York Heart Association; PND = paroxysmal nocturnal dyspnea; RWT = relative wall thickness; SAM = systolic anterior motion; SR = sinus rhythm; TR = tricuspid regurgitation; T2W FLAIR = T2-weighted-fluid-attenuated inversion recovery.

## Data Availability

Data are available upon request from the authors.
